# Identification of Athleticism and Sports Profiles Throughout Machine Learning Applied to Heart Rate Variability

**DOI:** 10.3390/sports13020030

**Published:** 2025-01-22

**Authors:** Tony Estrella, Lluis Capdevila

**Affiliations:** 1 Sport Research Institute, Universitat Autònoma de Barcelona, 08193 Bellaterra, Spain; antonio.estrella@uab.cat; 2 Laboratory of Sport Psychology, Department of Basic Psychology, Universitat Autònoma de Barcelona, 08193 Bellaterra, Spain

**Keywords:** heart rate variability, machine learning, athletes, sport profiles, team sports, random forest, support vector machine, SHAP values, training load

## Abstract

Heart rate variability (HRV) is a non-invasive health and fitness indicator, and machine learning (ML) has emerged as a powerful tool for analysing large HRV datasets. This study aims to identify athletic characteristics using the HRV test and ML algorithms. Two models were developed: Model 1 (M1) classified athletes and non-athletes using 856 observations from high-performance athletes and 494 from non-athletes. Model 2 (M2) identified an individual soccer player within a team based on 105 observations from the player and 514 from other team members. Three ML algorithms were applied —Random Forest (RF), Extreme Gradient Boosting (XGBoost), and Support Vector Machine (SVM)— and SHAP values were used to interpret the results. In M1, the SVM algorithm achieved the highest performance (accuracy = 0.84, ROC AUC = 0.91), while in M2 Random Forest performed best (accuracy = 0.92, ROC AUC = 0.94). Based on these results, we propose an athleticism index and a soccer identification index derived from HRV data. The findings suggest that ML algorithms, such as SVM and RF, can effectively generate indices based on HRV for identifying individuals with athletic characteristics or distinguishing athletes with specific sports profiles. These insights underscore the importance of integrating HRV assessments systematically into training regimens for enhanced athletic evaluation.

## 1. Introduction

Recovery in sports is regarded as a multifaceted, physiological and psychological, restorative process relative to time [1]. Fatigue is a condition of augmented tiredness due to physical and mental effort, and it can be compensated with recovery to re-establish a balanced state [2]. Thus, recovery is an essential process to both prevent physical injuries and improve stress management [3]. For athletes, an adequate balance between stress and recovery is essential to achieve continuous high-level performance [1,3]. In the field of physical preparation integrated into sports training, internal load (IL) and external load (EL) are distinct yet complementary measures used to evaluate an athlete’s adaptation to effort [4]. Its evaluation offers the possibility of adjusting the loads on a day-to-day basis [5] with the objective of reducing the risk of injury to players during the season [4,6].

A key parameter currently for measuring IL in sports is Heart Rate Variability (HRV) [7], which is considered an effective tool for monitoring adaptation to daily loads and training programs [8,9,10]. Several studies indicate the importance and usefulness of HRV evaluation to plan the training load and avoid overtraining [11], as well as the interest in analysing HRV in the prevention of fatigue states [12], or the quantification of the training load and the effect it has on sports performance [13]. Usually, HRV analysis consists of a standardised 5 min test at rest in a supine position [7]. The same test is used in the general population as a biomarker of athletic fitness related to health and physical performance [14]. Thus, athletes typically exhibit better cardiac autonomic function, characterised by greater heart rate variability, compared to non-athletes [15,16].

On the other hand, data analysis in sports has evolved with the growth of data science, particularly with the advancement of machine learning (ML) algorithms, enabling the integration of various types of data [17,18]. ML algorithms are generally classified into supervised learning and unsupervised learning. In supervised learning, the goal is to train an algorithm to predict outcomes when presented with new unseen data. To achieve this, the algorithm is trained using labelled data, where each observation in the dataset is associated with an outcome measurement. In contrast, unsupervised learning involves data that are not labelled, meaning there is no associated outcome measurement. The objective in this case is to discover patterns within the data [19,20].

ML algorithms offer a new approach, generating insights from data without requiring the imposition of a previous structure [21]. A key advantage over conventional data analysis is their flexibility, allowing them to handle non-linear relationships between features and outcomes [21]. However, a major limitation of ML algorithms has been their lack of interpretability [22] often regarded as “black-boxes”. Various explainability algorithms, such as SHapley Additive exPlanation (SHAP) values [23] or Local Interpretable Model-agnostic Explanations (LIME) [24], have emerged to address this concern, transforming these black-boxes into transparent models. SHAP values have been applied in the field of sports to identify athletic characteristics, such as growth factors in pre-adolescents [25]. Furthermore, SHAP values have been utilised to determine the contribution of performance indicators in a real-time model for predicting NBA game outcomes [26] and to identify key performance metrics across 11 elite football leagues in European countries [27]. This approach effectively translates model results into actionable insights for decision-making in sports [28].

Returning to the HRV analysis of our interest and regarding recovery behaviours, it has been found that it is a valuable parameter for classifying sleep stages closely linked to nocturnal recovery [29,30]. In the field of sports, the main role of HRV has been as a biomarker for classifying fatigue [31,32,33]. Additionally, resting HRV has been identified as a potential feature for estimating VO2max [34]. In summary, its usefulness in the field of team sports has been well justified, but it also has limitations [35]. For example, a difficulty encountered is that the interpretation of results from HRV tests for resting situations is based on the complex interaction of parameters that complicate the analysis to understand athletes’ health and performance [7]. This may cause some results to not be replicable, also due to the difficulty involved in monitoring control variables that may affect HRV analysis. Although it has been shown that HRV analysis provides specific values for elite athletes significantly different than in healthy controls, it has been noted that further investigations are needed to determine its role in the optimisation of training or identifying overtraining [36]. On the other hand, the use of ML algorithms in cardiac and HRV data analysis has seen an increase in the number of publications [37]. For example, it has been demonstrated the usefulness of ML in real-time scenarios combining physiological data and powerful ML models to improve an individual’s comprehension of fitness levels and the requirements for adaptive training [38]. A review shows that over the last few years, there has been a growing use of ML techniques to help improve understanding of the athlete’s heart, mainly from ECGs. But this review also highlights gaps for future research such as the need to adjust the accuracies of ML models to help improve the effectiveness of interventions [39].

However, there are still few studies on ML applications to explain HRV patterns in sports from datasets coming from sufficient HRV individual recordings in elite athletes. This work aims to overcome some of the limitations of previous studies on HRV analysis by applying ML, overcoming traditional analysis methods and addressing challenges in sports performance monitoring. The general aim of the present study is to check and apply ML methods in HRV parameters obtained in athletes and non-athletes according to the same strict methodology. The specific objectives are (a) to obtain an athleticism index for identifying people with athletic characteristics; and (b) to identify individual sports profiles for differentiating or recognising an athlete from the set of athletes who practice the same sport. We hypothesize that we will obtain specific ML algorithms for each objective: (a) an athleticism index based on the contribution of HRV parameters, both in the time and spectral domains, for calculating specific levels of physical fitness; (b) an individual sports index, based mainly on HRV spectral parameters, for differentiating a specific soccer player from the rest of the players on his team.

## 2. Materials and Methods

### 2.1. Study Sample

The study sample was a dataset containing 1350 recordings from 5 min HRV tests performed all at rest in the supine position in the same conditions. These recordings were obtained during 5 sports seasons and academic courses from 331 participants, of which 141 were high-performance athletes (78 soccer players, 37 basketball players, and 26 field hockey players; n = 856 HRV recordings) and 190 were healthy non-athletes (university students; n = 494 HRV recordings). All athletes trained daily and competed in the highest national and international categories. The soccer players belonged to the same club that was always ranked among the top two teams in the top state league and always qualified for the final phase of the European Champions League. No participants were excluded after being asked if they suffered from any of the following symptoms: physical abnormalities related to heart conditions, high blood pressure or taking heart medication, bone and joint problems, chest pain during activity, chest pain at rest, loss of balance, or dizziness. The age range was between 18 and 30 years old (mean = 24.45; SD = 4.69; 201 men and 130 women). All participants were volunteers, provided written informed consent to participate in the study and agreed to be sampled on several occasions by sports physicians. The protocol has been reviewed and approved by The Commission on Ethics in Animal and Human Experimentation (CEEAH) of the Autonomous University of Barcelona (protocol code CEEAH-5745; approved date 23 July 2021).

### 2.2. Procedure and Measures

**Figure 1:**
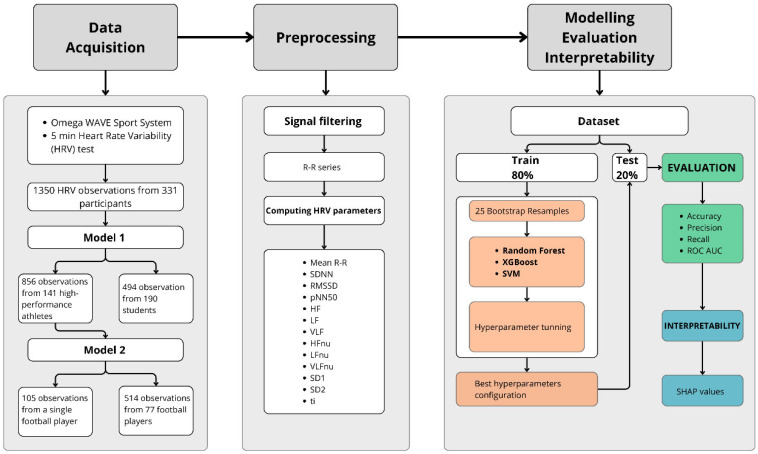
Machine learning workflow, spanning from data acquisition to interpretability.

Athletes were evaluated just before several trainings and students were evaluated just before university lessons. HRV assessment was performed usually in the mornings in a semi-dark room maintained at a comfortable temperature. After five minutes of rest lying down on a mat on the floor, participants were asked to remain supine and still without speaking or making any movements, and HRV data were registered continuously for five minutes of natural breathing. Before the sessions, all participants received information about specifications related to some variables, which could affect HRV analysis. Specifically, they were asked to avoid strenuous physical activity, caffeinated and alcoholic beverages, and taking nonessential medicines in the 24 h prior to the session, to avoid smoking and eating a heavy meal in the 3 h prior to the session, and to sleep at least 6 h the night prior to the session. Athletes always perform the HRV test before the training sessions. HRV data were collected using Omega Wave Sport System (Omegawave Oy, Espoo, Finland), with a resolution of 2 ms. The Omega Wave Sport System has been validated for detecting RR intervals [40] (Figure 1). This system uses a three-lead Electrocardiogram (ECG) which is connected to a computer by a transceiver box responsible for digitising the signal for processing within the computer. The digitised signal was filtered, in accordance with common standards for ECG readings, and processed by the Omega Wave Sport System software (Win 7 version) to obtain the R-R series. In order to obtain optimal ECG signal quality, artefacts in RR intervals were identified and corrected automatically prior to analysis as described in previous works [40]. The calculation of these HRV indices is consistent with the recommendations of the Task Force of the European Society of Cardiology and the North American Society of Pacing and Electrophysiology (1996) [7]. The HRV parameters used in this study are presented in Table 1.

### 2.3. Data Preparation

This study presents 2 different models with varying objectives. In model 1 (M1), the objective was to distinguish athletes and non-athletes (students), with 856 observations from athletes and 494 from students. In model 2 (M2), the goal was to identify a specific soccer player within the subsample of soccer players from the same M1 dataset. In M2,105 observations came from the individual player and 514 from the rest of the team.

In both models, the data were divided into 80% training and 20% testing, stratified by the variable of interest to maintain the same proportion in both parts (Figure 1). To prevent the model from being biased towards the majority class, the dataset in each model was balanced using the synthetic minority oversampling technique (SMOTE). SMOTE creates synthetic samples from the existing minority class through interpolation from its nearest neighbours, thereby increasing the number of minority samples in the datasets [41]. The number of neighbours was set at 10.

Furthermore, the search for the best hyperparameters was conducted on 25 bootstrap samples (Figure 1). Bootstrapping is a resampling method that repeatedly draws random samples with replacements from the original sample [42]. In these samples, the grid search method was employed, which considers all possible combinations of hyperparameters for each algorithm, selecting the best combination based on the area under the ROC curve (AUC-ROC) as a performance metric. The AUC-ROC metric ranges from 0 to 1, where a value of 1 represents a perfectly accurate classifier. In addition to selecting the optimal algorithm, accuracy, precision, and recall were chosen as performance metrics. These metrics also range from 0 to 1, with a score of 1 indicating a perfect classification.

### 2.4. Machine Learning Analysis

For this study, the supervised machine learning algorithms Random Forest (RF), extreme gradient boosting (XGBoost), and Support Vector Machine (SVM) were selected. These algorithms were deemed suitable for addressing HRV-related problems because they go beyond linear assumptions, offering flexibility by avoiding the imposition of a fixed structure [21,43]. This adaptability enhances their ability to generalize effectively to new data [19,44]. RF is an ensemble learning algorithm used for both regression and classification problems. It is based on the bagging method, although its main advantage over bagging is that RF considers only a set of predictors in each tree, thereby reducing the impact that some predictors may have on the model. This ensures that the trees in the RF are not correlated, making them more reliable [45]. RF is a highly popular algorithm that has been applied to various health-related problems and different types of data. XGBoost is a boosting algorithm that operates similarly to RF, except that its trees grow sequentially using information from the previous tree [46]. SVM was initially designed for binary and linear classification. It divides the observations using a hyperplane and calculates the distance of the nearest observations from it, with the closest observation referred to as a support vector. However, many problems require some non-linear solutions. To address this, SVM transforms the feature space by applying functions to the predictors (e.g., quadratic and cubic terms) [19]. To interpret the results of the ML algorithms for each model, SHapley Additive exPlainations (SHAP) values were used. SHAP values are based on cooperative game theory, where each feature of the model represents a player and their interactions towards the overall outcome [23]. All analyses were performed using the open-source software RStudio version 4.2.2 [47]. The R packages used were tidyverse [48] for exploratory data analysis, and tidymodels [49] for data modelling. All code is available at https://github.com/estrellatonyy/HRV_ML_analysis.git the creation date 19 September 2024.

## 3. Results

In this Section, descriptive parameters for each model and the steps followed in the ML process are outlined. First, model evaluation with hyperparameters tunning was performed for each algorithm to select the configuration that best fits the data. Next, the optimal configuration was applied to each model and was evaluated in terms of performance metrics. In addition, the best solution generated was interpreted using SHAP values. Finally, a predictive index is proposed for each model.

### 3.1. Descriptive Parameters

In M1 a total of 1350 observations were included, 494 from university students and 856 from high performance athletes. Table 2 shows all HRV initial parameters included in ML calculations for M1.

In M2 a total of 619 observations were included, 105 of them from a single soccer player, and 514 from the soccer team to which the soccer player belongs. HRV initial parameters for M2 are outlined in Table 2.

### 3.2. Machine Learning Evaluation

Hyperparameters tunning was performed on 25 bootstrap samples using a maximum entropy design, which identifies a configuration of points that effectively covers the parameter space while minimising overlap and redundancy [49,50]. For RF algorithms, the selected hyperparameters included were the number of sampled predictors, the number of trees, and the number of data points required for a split. For the XGBoost model, the same hyperparameters as the RF algorithm were used, with the addition of the learning rate. For SVM, the cost and radial basis function sigma were selected. The optimal hyperparameter combinations for each algorithm are presented in Table 3. Consult Supplementary Materials for a further description about the hyperparameter tuning process.

Regarding model evaluation with the test data, in M1 the algorithm that performed best was SVM (Cost = 20.74; Radial Basis Function sigma = 0.00012), with an accuracy of 0.84 and a ROC AUC of 0.91. Tree-based algorithms performed slightly worse. The RF algorithm achieved an accuracy of 0.82, with a ROC AUC of 0.90. XGBoost showed the lowest performance in terms of accuracy and ROC AUC (0.80 and 0.89, respectively). Table 4 presents the performance metrics for each algorithm. Although the three algorithms produced different results, overall performance in M1 was very good for athlete classification.

In M2, the RF algorithm (number of sampled predictors (mtry) = 2; number of trees (trees) = 214; number of data points to split (min_n) = 10) achieved the highest performance, in terms of accuracy (0.93). However, the XGBoost achieved the highest ROC AUC (0.95). In contrast, the SVM showed the lowest performance, with an accuracy of 0.81, and a ROC AUC of 0.92. Table 4 presents the results for these algorithms in M2. In this model, tree-based algorithms outperformed the SVM.

### 3.3. Interpretability with SHAP Values

**Figure 2:**
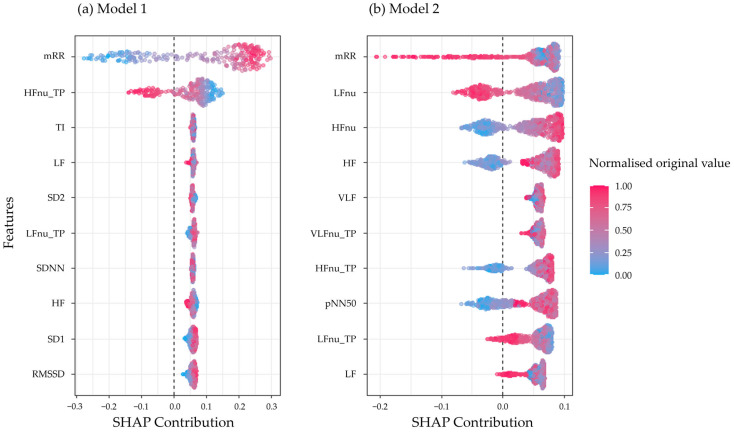
SHapley Additive exPlanation (SHAP) values ranking the top 10 HRV parameters contributing to the model: (**a**) SHAP val ues for Model 1. (**b**) SHAP values for M2.

SHAP values have been applied to RF algorithms with hyperparameter tunning for the M1 and M2. The contribution of the HRV parameters for each model is shown in Figure 2. Explanatory variables are ordered from the most to least contributor on the *y*-axis. The top 5 HRV parameters are based on their average absolute contribution to the target. On the *x*-axis indicates the SHAP value expressed as the change in the log odds, resulting in a positive or negative contribution for a specific observation. Each dot represents an observation in the dataset and the colour is indicative of the original value for that observation, with high values displayed as red and low as blue. The vertical dotted line represents when the SHAP contribution is zero, the right side assigns a positive contribution and the left side a negative contribution.

In M1, the five HRV variables with the highest contribution are mRR, HFnu_TP, TI, LF, and SD2. The mRR parameter indicates that higher values are associated with positive athlete classification, contributing up to 0.3 SHAP value. In other words, higher mRR values are linked to the athlete category. In M2, the five HRV parameters with a higher contribution to identifying a soccer player are mRR, LFnu, HFnu, HF, and VLF. Since this is a different model, the interpretation changes, higher mRR and LFnu values, contribute negatively to player identification, while higher HF and HFnu values contribute positively to identifying the target player.

The HRV parameter mRR was the most significant contributor in both models, and along with TI in M1, was one of the only time domain variables among the top 5 contributors. In contrast, the frequency domain HRV parameters appeared in both models.

### 3.4. Athleticism Index

**Figure 3:**
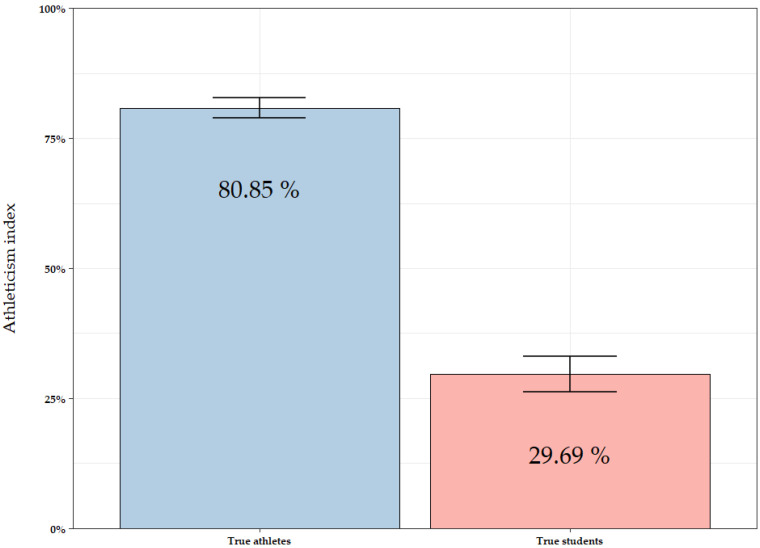
Average athleticism index assigned by the Support Vector Machine algorithm to true athletes (blue) and true students (red) in the test set. Error bars represent the standard errors.

In this Section, an athleticism index derived from M1 is proposed based on the prediction performance of the SVM algorithm in the test data. Figure 3 shows a bar chart illustrating the mean value of the athleticism index assigned to true athletes (blue) and true students (red) in the test set. The SVM algorithm predicts an average athleticism index of 80.85% for athletes and 29.69% for students. The index represents the proportion of athletics characteristics identified in each group. The overall performance of the SVM algorithm, as depicted in Figure 3, demonstrates its effectiveness in distinguishing between athletes and non-athletes in the test sample. Therefore, based on these results, the proportion used to classify athletes is proposed as an athleticism index.

**Figure 4:**
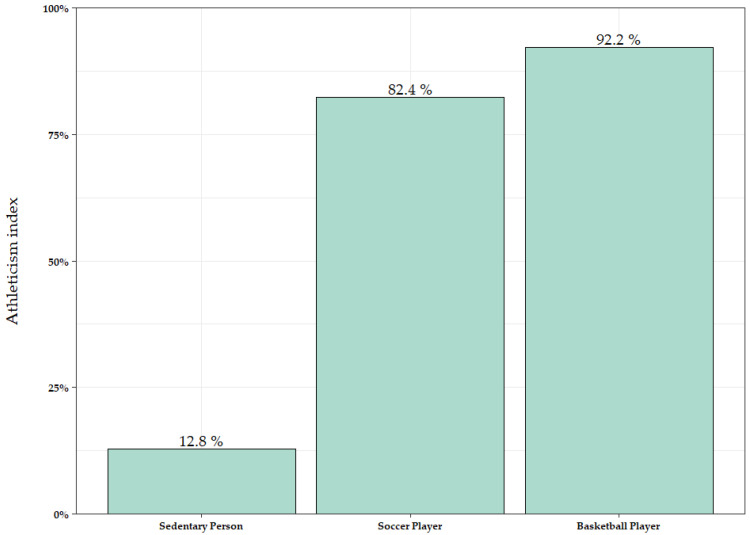
Athleticism index for new predictions in Model 1 across different groups: Sedentary person, soccer player, and basketball player.

The SVM algorithm in M1 was applied for the prediction of three distinct HRV tests performed on new participants who were not included in the initial sample of the study: one from a sedentary (non-athlete) participant, one from a soccer player, and one from a basketball player. For the sedentary participant, the algorithm predicted a probability of 0.128 (12.8%) for being classified as an athlete. For the soccer player, the predicted probability of being classified as an athlete was 0.824 (82.4%). Finally, for the basketball player, the probability of being classified as an athlete was 0.922 (92.2%) (Figure 4). Table 5 shows the HRV parameters for each new observation introduced in the model.

### 3.5. Soccer Identification Index

In this Section, an individual soccer identification index derived from M2 is proposed based on the prediction performance of the RF algorithm. This identification index represents the probability of correctly identifying a specific soccer player. A high identification index indicates a strong prediction, while a low identification index reflects a weaker prediction.

**Figure 5:**
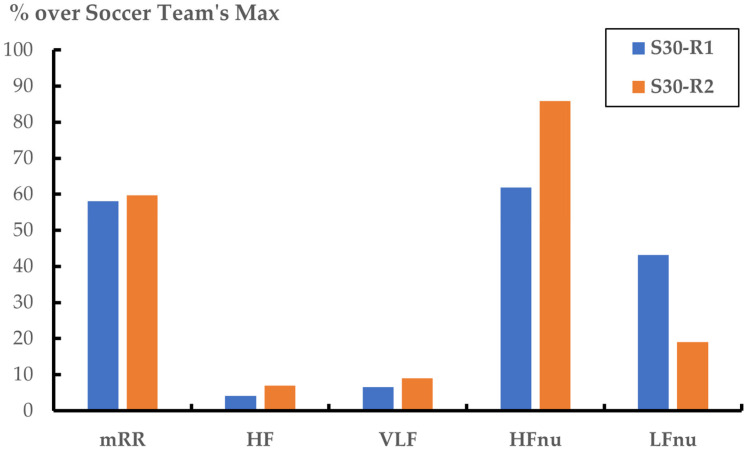
Comparison of two HRV test observations through the percentage of the 5 top parameters provided by the best ML algorithm (RF) in M2, calculated with respect to the maximum value observed in the soccer team. S30-R1: HRV test observations where the soccer player S30 is misclassified (0.2044). S30-R2: HRV test observations where the soccer player S30 is almost perfectly identified (0.9924).

Regarding M2, the RF algorithm demonstrated strong predictive performance, with evaluation metrics exceeding 0.9 (Table 4). In this model, the resulting index makes it possible to identify an individual soccer player (S30) from the rest of his soccer team. Thus, we use the HRV parameters depicted in Table 5 to calculate the soccer identification index for each observation (S30-R1 and S30-R2). Figure 5 illustrates the two test observations, one in which the player is almost perfectly identified (S30-R2: 0.9924) and another where the model misclassifies him (S30-R1: 0.2044). The comparison between these two records in Figure 5 is based on the five HRV parameters identified by the SHAP value algorithm as top contributors in M2. Each parameter’s percentage is calculated with respect to the maximum observed value within the soccer team. As shown in Figure 5, all HRV parameter percentages are higher in R2 than in R1 except for LFnu, with an inverse relationship observed in the parameters HFnu and LFnu.

## 4. Discussion

This study employed a novel approach to identify athletic and sports-related characteristics based on a simple and non-invasive HRV test at rest. Time domain, frequency domain, and non-linear parameters from HRV analysis were computed to determine these characteristics. Two distinct models were developed: Model 1 (M1) aimed to distinguish between athletes and non-athletes proposing an athleticism index, while Model 2 (M2) sought to identify a specific soccer player from the subsample of soccer players in the M1 dataset. This contribution is supported by the high quality of the data used in the models. In this study, we carefully selected methods to assess athletes over five seasons, prioritising quality over quantity to build robust ML models [51]. It should be noted that all HRV recordings were performed individually and strictly following the procedure described in Section 2. For calculating each individual’s HRV parameters, we consistently began with the raw data composed of all interbeat intervals (IBI or RR intervals), applying the same filtering process to correct any RR registration error. In other words, we have always relied on the same calculations from the original IBIs, and not on HRV parameter values from unknown databases. The results for M1 and M2 show that Support Vector Machine (SVM) and Random Forest (RF) algorithms achieved an accuracy of 84% (0.84) and 92% (0.92), respectively (accuracy values range from 0 to 1, where 1 is the maximum, and it can be interpreted as a percentage). According to the results of these models, it is possible to identify individual athletic and sports-related characteristics.

Additionally, an athleticism index is proposed in M1 derived from the high performance of the SVM algorithm. The resulting index provides a percentage of athleticism indicating the likelihood that an individual belongs to the athlete group. It should be noted that this index is tested using new individual HRV test recordings, assigning a probability of classification with respect to the target group. In our study, we applied the SVM algorithm in M1 for the prediction of three distinct HRV tests collected from new participants who were not included in the initial sample of the study. The first is a non-athlete, a healthy but non-physical active person who was not included in the initial dataset and who performed an HRV test following exactly the same procedure indicated in Section 2. The SVM algorithm calculates an athleticism index of 12.8% (0.128) or, which is the same, a probability of 87.2% that the individual is a non-athlete. This result suggests that the algorithm accurately predicts a low level of physical fitness for this person. The second new participant, a soccer player, has an athleticism index of 82.4%, with a probability of 17.6% of being a non-athlete. The third new participant, a basketball player, shows an athleticism index of 92.2%, with a 7.8% probability of being a non-athlete. We can interpret in both cases that the SVM algorithm also correctly predicts that these two persons have a high level of physical fitness, probably derived from their daily workouts. In addition, we could also interpret that the basketball player has a higher training load overall than the soccer player, to in accordance with the idea that basketball players present an absolute total energy expenditure greater than that of soccer players [52].

Therefore, the athleticism index proposed could serve as a training load measure in athletes or a fitness indicator in non-athletes, based on HRV analysis and ML algorithms. Remember that in M1 the five HRV parameters that achieved higher contributions to classify athletes were mRR, HFnu_TP TI, LF, and SD2. Particularly, mRR and HFnu_TP were the most contributive HRV parameters, probably reflecting the effort recovery characteristic of athletes. These results align with previous research which highlighted that athletes exhibit higher vagal tone that contributes to a lower resting heart rate [53]. Furthermore, another study that compared the HRV values between athletes and sedentary subjects found that HF was one of the most important parameters for measuring the athletes’ health status [54]. However, in M1, the HFnu_TP parameter could be interpreted as higher values contribute negatively to the identification of athletes, as Table 2 shows as well. This result could be because elite athletes are subjected to continuous high-intensity training sessions that could affect the involvement of HF in the recovery of the effort [55]. However, a different study which aimed to predict athletic performance in anaerobic sprinters found that the HRV parameter with higher relative importance was RMSSD [56]. These differences with the present study might be due to the use of different time duration registers (24 h vs. 5 min) [53], and the use of the relative importance index instead of the SHAP value algorithm.

In the case of M2, we compared two HRV test recordings for the same soccer player (S30), corresponding to the 20% of them reserved for analysing the testing or predictive performance. He is a soccer player coming from the base soccer school of the club to which the soccer team belongs and with more than 10 years of experience playing in the highest category according to his age. He performed a total of 105 HRV tests, included in the dataset, following the same procedure indicated in Section 2. In M2, we interpret the results so that applying the RF algorithm to the two specific HRV tests yields different outcomes: the first one (R1) identifies the soccer player S30 with a 20.4% among the team members, while the second HRV test (R2) identifies him with a 99.2%. This identification index proposed in M2 could have potential application from the performance of daily and regular HRV tests, particularly during the pre-season phase when athletes are subjected to higher external load training than during the in-season [57,58]. In M2, the five most contributive HRV parameters were mRR, LFnu, HFnu, HF, and VLF. Apart from RRmean, in this model, the frequency parameters have an important role in identifying the specific soccer player. It could serve as a useful tool for assessing readiness, how well athletes are adapting to training loads, or how external load affects the individual internal loads [2]. In this sense, HRV analysis has been proposed as an adequate index of internal load [59], which is sensitive to changes in the athletes’ state. Specifically, the index misclassifies the soccer player when significant deviations occur in their internal load. Likewise, the usefulness of HRV analysis to assess the level of fitness in a non-athlete population has also been indicated [15]. Thus, the index found in M2 could be applied as an internal training load index for monitoring both acute and chronic training loads for an individual player in a team sports context. This index could be integrated into daily training routines and used when there is sufficient team data from previous HRV tests, as in the aforementioned example of the soccer player S30. Only in this case could the index obtained from a simple HRV test before a specific workout have the potential for real-time feedback in athlete monitoring. This may provide valuable insights for training smarter and for preventing training-related injuries by tracking how an athlete’s HRV responds to different training and competition loads [60]. However, a limitation of our study is the difficulty in generalising the results to other team sports. It is possible that the ML algorithm is different, that it does not fit the data equally well, and that the HRV parameters that compose it are different. Future research could address this limitation by integrating daily HRV measurements into ML models. This would offer a more dynamic understanding of individual responses to training loads. By incorporating these daily HRV measurements, ML models could be further refined, generating more robust indices and personalised recommendations for athletes.

In this study, three classification algorithms were employed from two different families. The use of diverse algorithms is recommended due to the varying ways algorithms fit the data [61]. Despite all three algorithms showing strong performance in terms of evaluation metrics, SVM and RF achieved superior results in M1 and M2, respectively. These findings suggest that both ensemble methods (such as RF) and SVM are highly effective for HRV data, implying that more complex models may not be necessary in this context. HRV data are considered mostly non-linear, consequently, the use of non-linear algorithms, such as those used in this study, improves their application in a real-life situation [62,63]. These results are consistent with previous research that predicted subjective exertion during endurance training by combining Inertial Measurement Units (IMU) and HRV features, where SVM, RF, and XGBoost were similarly used [64]. Additionally, another study on physical exertion during exercise found that RF outperformed neural networks and linear regression in classifying cardiorespiratory data [65]. However, our study has a limitation related to the class imbalance in each model, which was addressed using SMOTE. While SMOTE is an effective technique for handling imbalanced datasets, it is not without its limitations. SMOTE can potentially bias the model performance by overfitting the minority class. This issue is particularly pronounced in datasets containing noise, as synthetic observations may replicate these artefacts. To mitigate this risk in our study, the dataset was filtered and cleaned prior to applying any processing step.

A comparison of results between M1 and M2 showed that not only did the hyperparameters need adjustment, but the overall algorithm performance shifted as well. This suggests that the target outcome, group classification in M1 (athletes vs. non-athletes) versus individual identification in M2 (soccer player), significantly impacts the algorithm’s performance, even when using the same type of HRV data [66]. These differences underscore the importance of selecting and tuning algorithms based on the specific problem being addressed.

## 5. Conclusions

As the main conclusion of our study, we can state that applying ML methods to HRV analysis can be a very useful tool for monitoring training loads in team sports. HRV analysis at rest can be a simple and non-invasive test to monitor how the external load in team sports affects the individual internal load. ML methods such as SVM and RF allow us to obtain indices for identifying people with athletic characteristics or athletes with specific sports profiles. This is only possible when we integrate rigorous and systematic HRV test recordings into daily training routines. Thus, tracking how athletes respond to different training and competition loads through a simple HRV test, can provide immediate feedback, enabling adjustments to individual training load if an extreme index is obtained when applying the ML algorithm. This may provide valuable insights for optimising training and preventing training-related injuries.

## Figures and Tables

**Table 1:** Description of time, frequency, and non-linear domain heart rate variability parameters.

Measure	Parameter	Units	Description
Time domain	Mean R-R	ms	Average duration of all R-R intervals (mRR).
	SDNN	ms	Standard deviation of all R-R intervals.
	RMSSD	ms	Square root of the mean of the sum of the squared differences of all RR intervals.
	pNN50	%	Percentage of consecutive RR intervals that differ by more than 50 ms.
	TI	ms	Triangular index (TI). Baseline width of the minimum square difference triangular interpolation of the highest peak of the histogram of all NN intervals.
Frequency domain	HF	ms ^2^	Power in high frequency (HF) range (0.15–0.4 Hz).
	LF	ms ^2^	Power in low frequency (LF) range (0.04–0.15 Hz).
	VLF	ms ^2^	Power in very low frequency (VLF) range (0.003–0.04 Hz).
	LF/HF		Ratio of low-to-high frequency.
	HFnu	n.u. ^1^	HF power in normalised units. HF/(Total Power–VLF) × 100.
	LFnu	n.u. ^1^	LF power in normalised units. LF/(Total Power–VLF) × 100.
	HFnu_TP ^2^	n.u. ^1^	HF power in normalised units. HF/(Total Power) × 100.
	LFnu_TP ^2^	n.u. ^1^	LF power in normalised units. LF/(Total Power) × 100.
	VLFnu_TP ^2^	n.u. ^1^	VLF power in normalised units. VLF/(Total Power) × 100.
Non-linear domain	SD1	ms	Standard deviation of the orthogonal intervals from RRi, RRi + a to the transverse diameter of the ellipse. Poincaré plot crosswise.
	SD2	ms	Standard deviation of the orthogonal intervals from RRi, RRi + a to the longitudinal diameter of the ellipse. Poincaré plot lengthwise.

^1^ Normalised units; ^2^ TP: Total power for spectral analysis (HF + LF + VLF).

**Table 2:** Description of all HRV parameters included in ML calculations, for time and frequency domains for each model.

Parameter	Model 1		Model 2	
	Students (non-Athletes)	Athletes	Soccer Player	Soccer Team
Mean R-R (ms)	902.54 (139.70)	1120.00 (163.11)	1060.87 (98.52)	1112.35 (165.26)
SDNN (ms)	72.59 (32.56)	78.12 (35.69)	79.47 (13.38)	75.01 (33.77)
RMSSD (ms)	73.28 (44.28)	81.58 (49.38)	85.22 (17.79)	76.85 (47.49)
pNN50 (%)	39.22 (22.84)	45.55 (22.33)	60.19 (10.39)	42.23 (23.00)
HF (ms^2^)	3795.96 (4554.67)	2705.26 (4278.90)	3243.41 (1445.94)	2394.53 (4167.77)
LF (ms^2^)	1069.78 (1518.45)	2020.43 (3663.65)	1069.57 (537.87)	1879.51 (3615.19)
VLF (ms^2^)	717.30 (1251.30)	1665.88 (2044.05)	1569.86 (1317.49)	1603.35 (1728.86)
LF/HF	0.63 (1.25)	1.37 (1.78)	0.40 (0.29)	1.59 (1.99)
HFnu (n.u.) ^1^	72.65 (20.24)	53.70 (20.51)	73.63 (12.05)	50.25 (20.27)
LFnu (n.u.) ^1^	27.35 (20.24)	46.30 (20.51)	26.37 (12.05)	49.75 (20.27)
HFnu_TP ^2^ (n.u.) ^1^	61.73 (22.53)	38.63 (19.31)	55.47 (14.76)	35.30 (18.89)
LFnu_TP ^2^ (n.u.) ^1^	21.74 (15.96)	31.71 (16.05)	19.52 (9.51)	33.10 (16.03)
VLFnu_TP ^2^ (n.u.) ^1^	16.53 (14.61)	29.66 (18.27)	25.01 (13.50)	31.60 (19.20)
SD1 (ms)	51.81 (31.31)	57.68 (34.91)	60.26 (12.58)	54.34 (33.58)
SD2 (ms)	87.76 (35.84)	92.80 (39.77)	94.39 (16.38)	89.52 (37.78)
TI (ms)	223.82 (97.13)	233.07 (115.35)	268.92 (81.76)	221.26 (97.96)

Mean (SD); ^1^ Normalised units; ^2^ TP: Total power for spectral analysis (HF + LF + VLF).

**Table 3:** Best algorithms’ hyperparameters configuration for each model.

Algorithm	M1	M2
RF ^1^	Number of sampled predictors (mtry) = 5; Number of trees (trees) = 429; Number of data points to Split (min_n) = 16	Number of sampled predictors (mtry) = 2; Number of trees (trees) = 214; Number of data points to Split (min_n) = 10
XGBoost ^2^	Number of sampled predictors (mtry) = 7; Number of trees (trees) = 219; Number of data points to Split (min_n) = 18; Learning rate (learn_rate) = 0.007	Number of sampled predictors (mtry) = 5; Number of trees (trees) = 469; Number of data points to Split (min_n) = 17; Learning rate (learn_rate) = 0.00126
SVM ^3^	Cost = 20.74; Radial Basis Function sigma = 0.00012;	Cost = 0.011; Radial Basis Function sigma = 0.054;

^1^ RF: Random Forest; ^2^ XGBoost: Extreme Gradient Boosting; ^3^ SVM: Support Vector Machine.

**Table 4:** Algorithms’ performance for each model.

	M1			M2		
Parameter/Algorithm	RF ^1^	XGBoost ^2^	SVM ^3^	RF ^1^	XGBoost ^2^	SVM ^3^
Accuracy	0.82	0.80	0.84	0.92	0.87	0.81
Precision	0.73	0.70	0.75	0.93	0.93	0.95
Recall	0.82	0.81	0.86	0.97	0.90	0.81
AUC-ROC	0.90	0.89	0.91	0.94	0.95	0.92

^1^ RF: Random Forest; ^2^ XGBoost: Extreme Gradient Boosting; ^3^ SVM: Support Vector Machine.

**Table 5:** Five top HRV parameters incorporated into the two best-performing ML algorithms, with 3 observations that were previously unseen by SVM in M1, and 2 observations from the test dataset for RF in M2.

Parameter	M1: SVM			M2: RF	
	Sedentary Person	Soccer Player	Basketball Player	S30-R1 ^1^	S30-R2 ^2^
Mean R-R (ms)	818.21	1056.9	1094.8	956.16	981.71
HF (ms^2^)				2350.26	3959.85
LF (ms^2^)	466.46	1423.8	2901.9		
VLF (ms^2^)				818.89	1107.81
HFnu (n.u.) ^3^				58.96	81.90
LFnu (n.u.) ^3^				41.04	18.10
HFnu_TP ^4^ (n.u) ^3^	21.94	39.11	38.99		
SD2 (ms)	37.3	57.96	81.91		
TI (ms)	148	226	377		

^1^ S30-R1: HRV test observations where the soccer player S30 is misclassified (0.2044). ^2^ S30-R2: HRV test observations where the soccer player S30 is almost perfectly identified (0.9924). ^3^ Normalised units; ^4^ TP: Total power for spectral analysis (HF + LF + VLF).

## Data Availability

The data presented in this study are available on request from the corresponding author. The data are not publicly available due to privacy and ethical restrictions.

## Supplementary Materials

The following supporting information can be downloaded at: https://www.mdpi.com/article/10.3390/sports13020030/s1, Figure S1: Hyperparameter search results for the Random Forest algorithm in Model 1; Figure S2: Hyperparameter search results for the Random Forest algorithm in Model 2; Figure S3. Hyperparameter search results for the Extreme Gradient Boosting algorithm in Model 1; Figure S4. Hyperparameter search results for the Extreme Gradient Boosting algorithm in Model 2; Figure S5. Hyperparameter search results for the Support Vector Machine algorithm in Model 1; Figure S6. Hyperparameter search results for the Support Vector Machine algorithm in Model 2.
