# Supplementary material for: Identification of Athleticism and Sports Profiles Throughout Machine Learning Applied to Heart Rate Variability
Source: Sports (Basel). 2025 Jan 22;13(2):30. doi: 10.3390/sports13020030 (PMC11860660; doi:10.3390/sports13020030)

Identification of athleticism and sports profiles throughout machine learning applied to heart rate variability

Supplementary Material

Tony Estrella and Lluis Capdevila

The hyperparameter search was conducted using a maximum entropy design to explore the parameter space efficiently. The y-axis represents the average value of the performance metrics, while the x-axis denotes the hyperparameter value. The dot color represents the mean value of the performance metric on a continuous scale, providing a clear visual summary of the results. The hyperparameter search was conducted in 25 bootstrap samples, and the performance metrics were averaged for each unique hyperparameter configuration.

Hyperparameters for each algorithm:

- Random Forest (RF): Number of sampled predictors (mtry), number of trees (trees), and number of data points to split (min_n). Figure S1 and Figure S2.
- Extreme Gradient Boosting (XGBoost): Number of sampled predictors (mtry), number of trees (trees), number of data points to split (min_n), and learning rate (learn_rate). Figure S3 and Figure S4.
- Support Vector Machine (SVM): Cost parameter, and the radial basis function kernel's sigma parameter. Figure S5 and Figure S6.

Figure S1. Hyperparameter search results for the Random Forest algorithm in Model 1

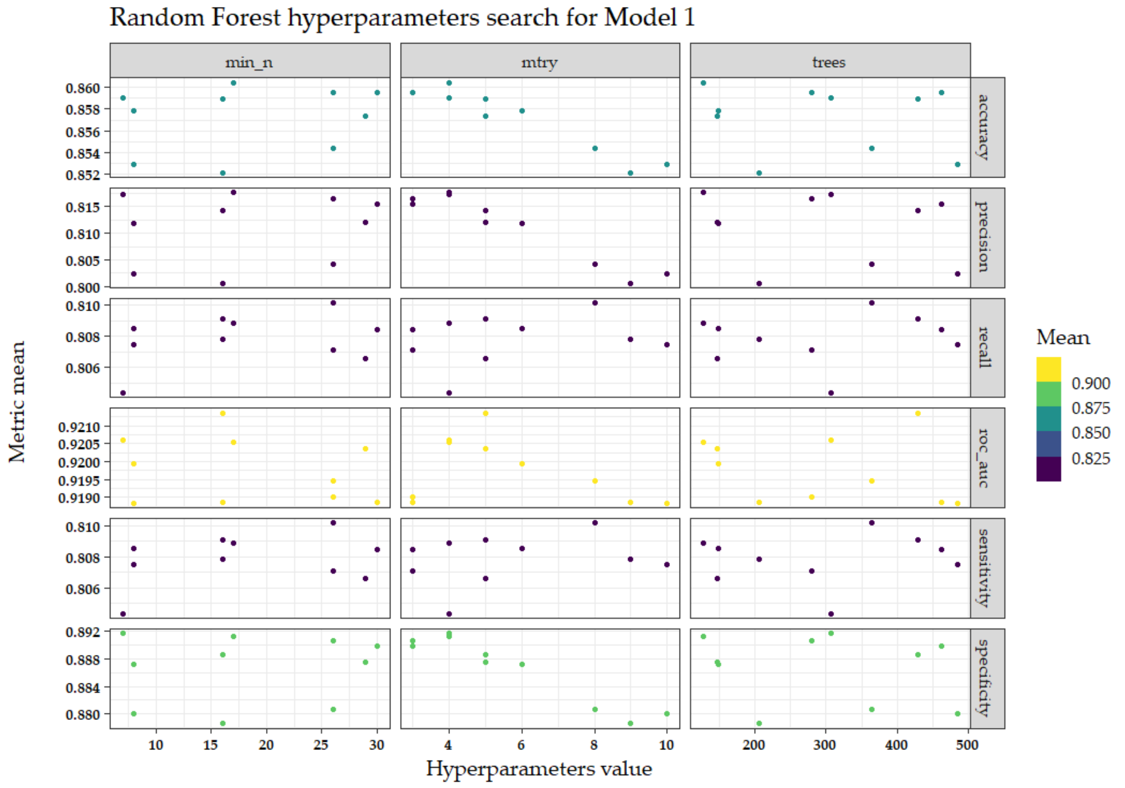

Figure S2. Hyperparameter search results for the Random Forest algorithm in Model 2.

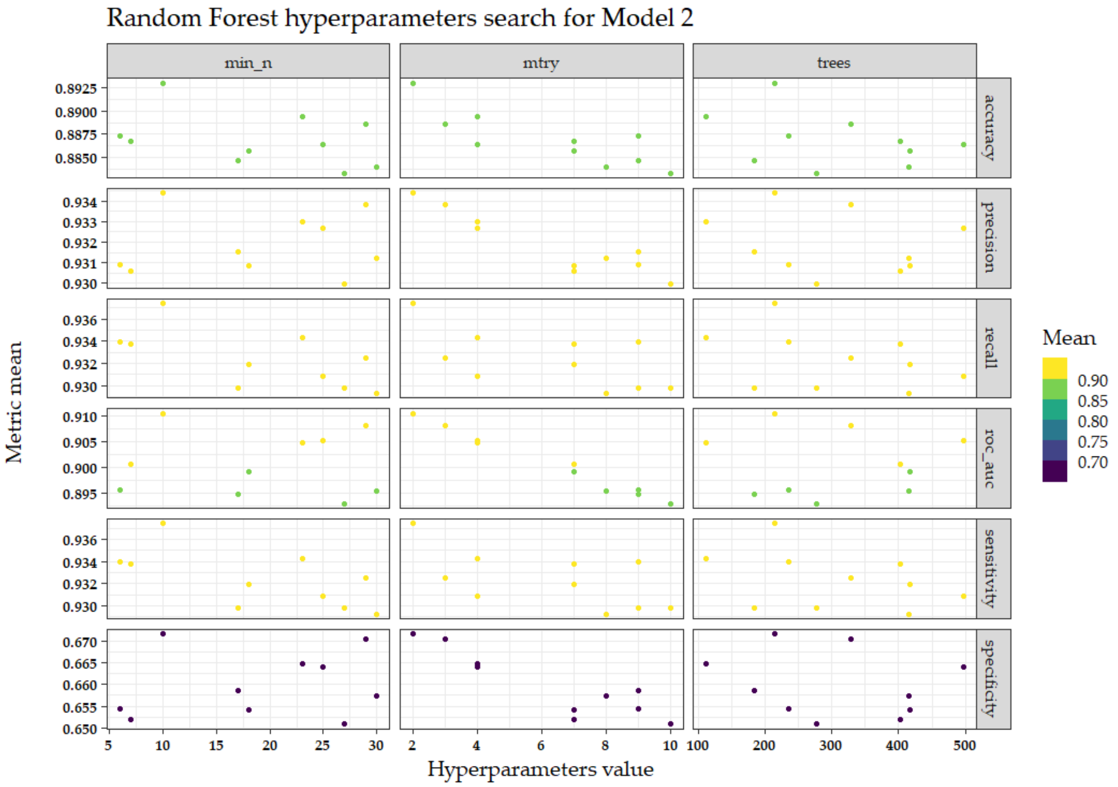

Figure S3. Hyperparameter search results for the Extreme Gradient Boosting algorithm in Model 1.

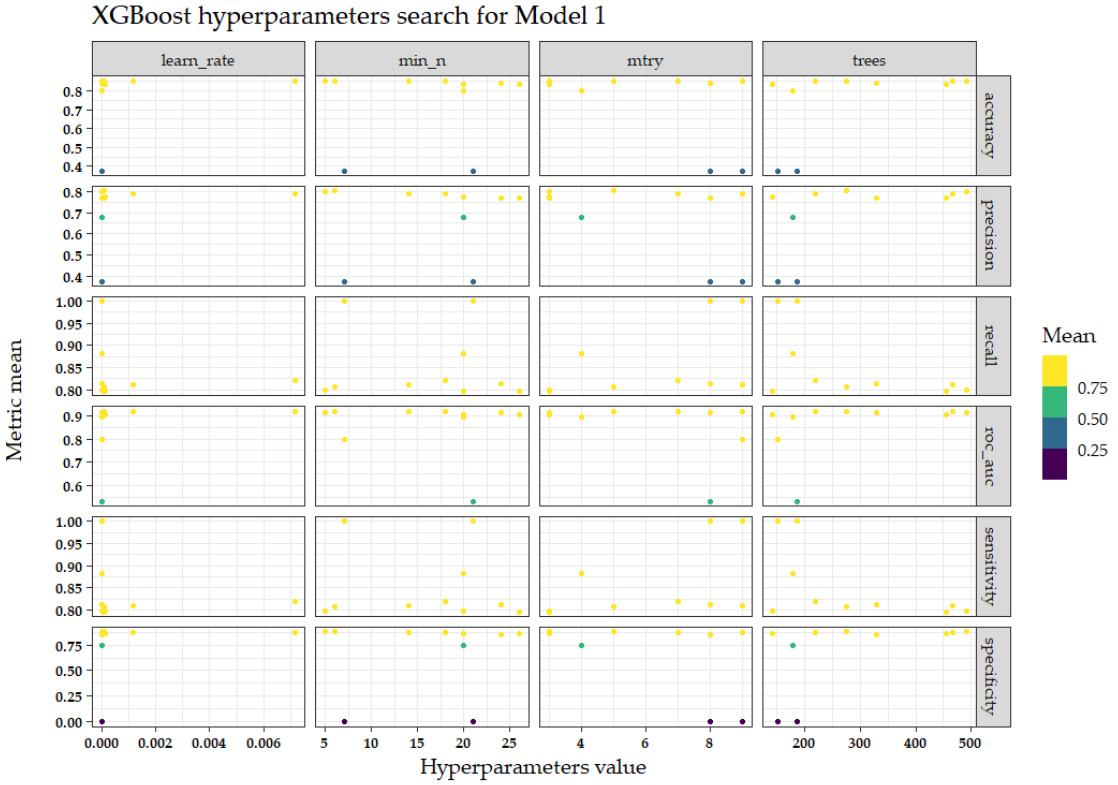

Figure S4. Hyperparameter search results for the Extreme Gradient Boosting algorithm in Model 2.

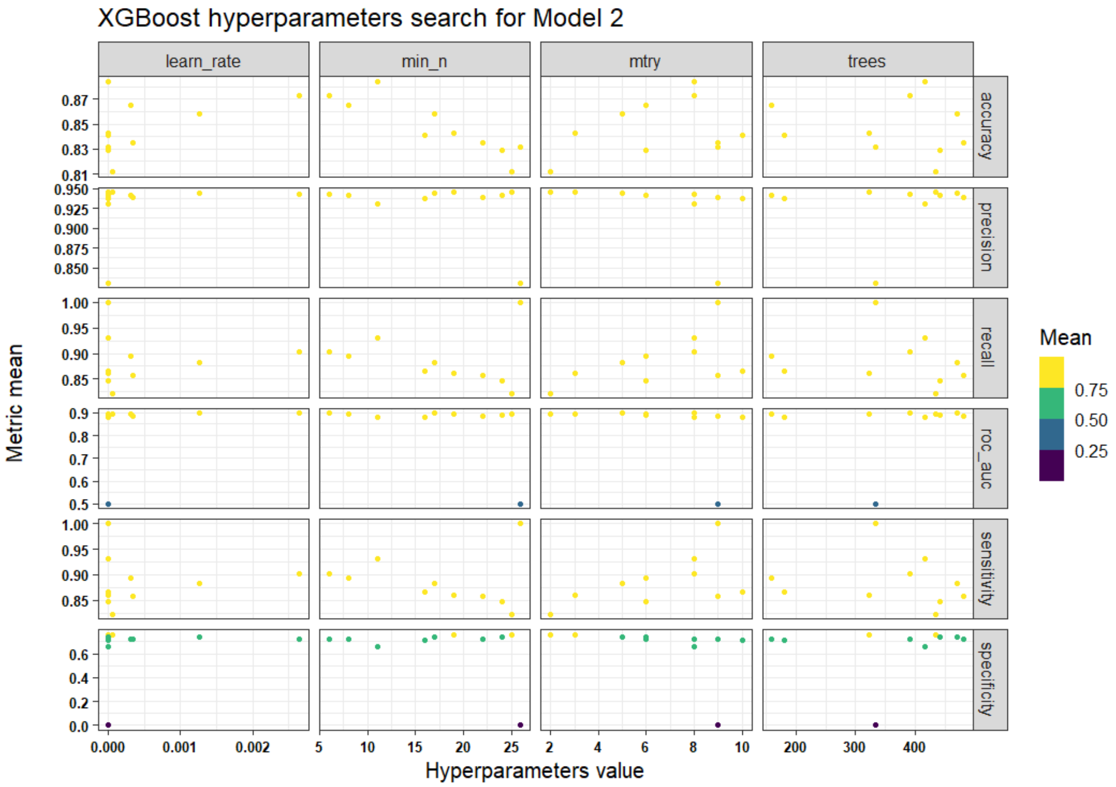

Figure S5. Hyperparameter search results for the Support Vector Machine algorithm in Model 1.

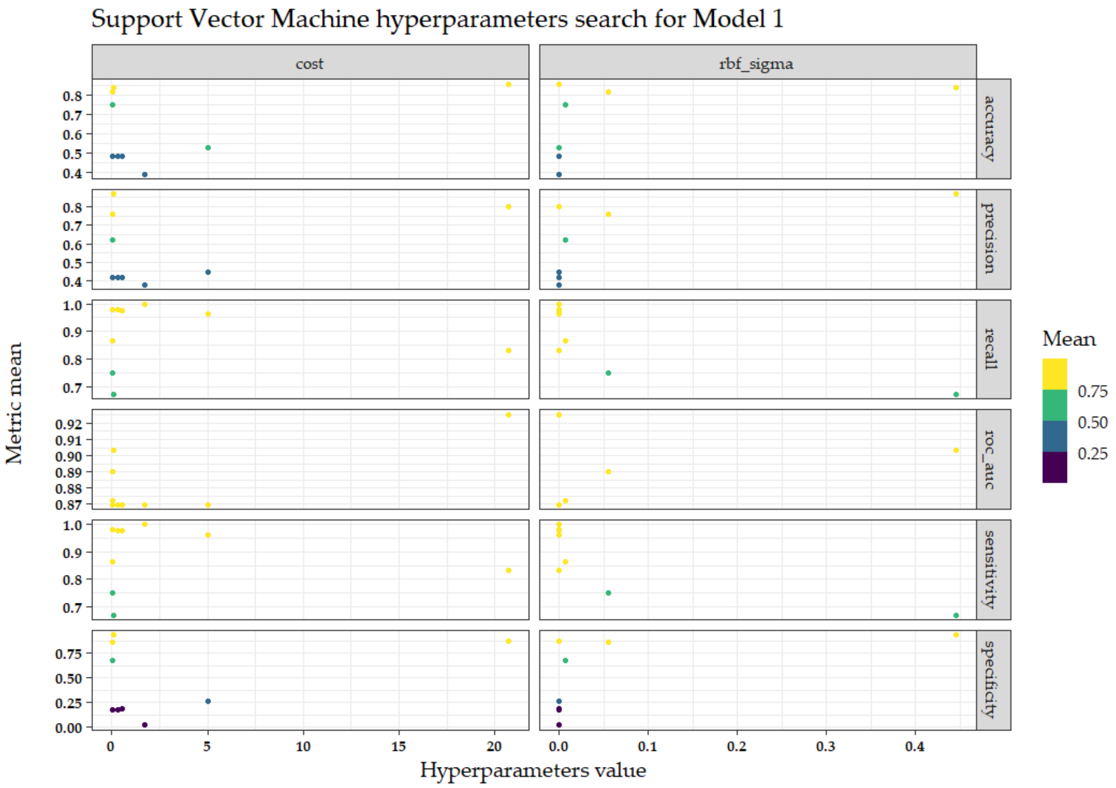

Figure S6. Hyperparameter search results for the Support Vector Machine algorithm in Model 2.

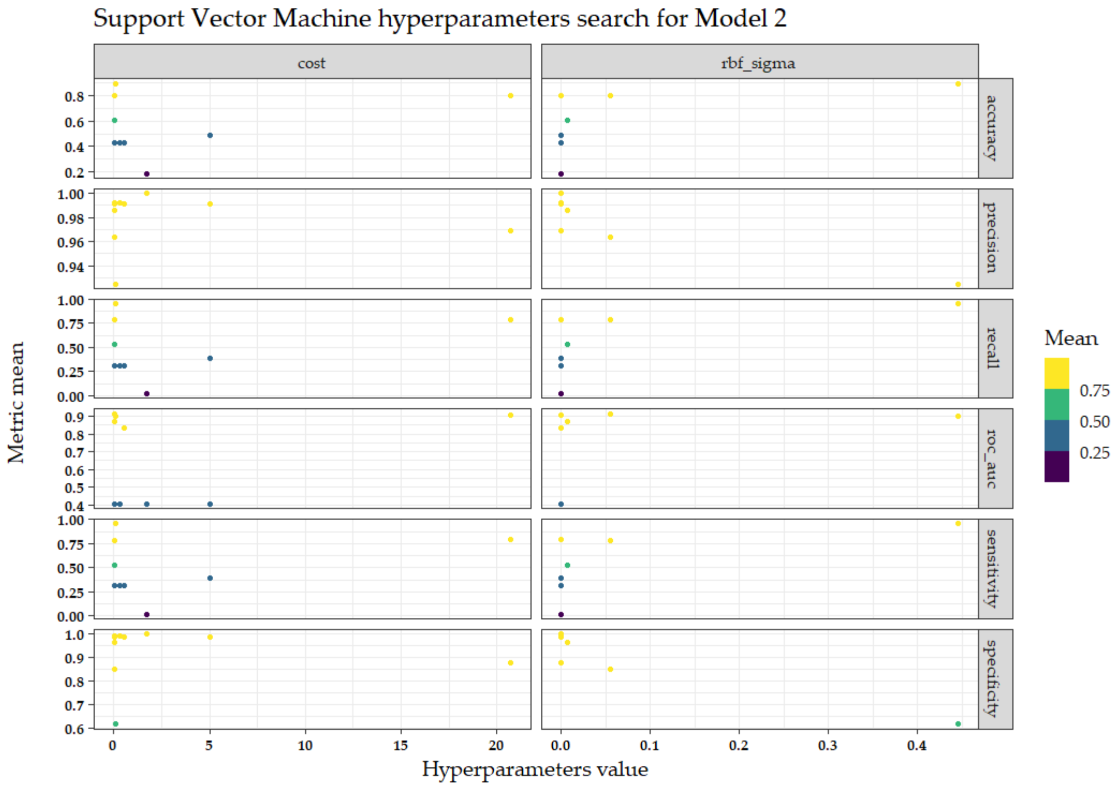

Supplement: Supplementary file 1 [file sports-13-00030-s001.zip › sports-3340975-supplementary.pdf]
